# Effects of Thermocycling with Two Different Curing Techniques on Enamel Micro-Cracks Formation, Debonding, and Failure Modes of Ceramic Brackets: An In Vitro Study

**DOI:** 10.3390/ma17153765

**Published:** 2024-07-31

**Authors:** Mohammad Qali, Lujain Alsulaimani, Nora Alamer, Faisal Alghamdi, Anwar Alhazmi, Ahmed Masoud

**Affiliations:** 1 Department of Surgical Sciences, College of Dentistry, Kuwait University, Kuwait City 13060, Kuwait; 2 Dental Department, Al Baha Specialized Dental Hospital, Ministry of Health, Al Bahah 65525, Saudi Arabia; lujainalsulaimanii@gmail.com; 3 Department of Periodontics and Community Dentistry, College of Dentistry, King Saud University, Riyadh 11421, Saudi Arabia; nalamer@ksu.edu.sa; 4 Department of Oral Biology, Faculty of Dentistry, King Abdulaziz University, Jeddah 80209, Saudi Arabia; falghamdi@kau.edu.sa; 5 Department of Preventive Dental Sciences, Dental College, University of Jazan, Jazan 45142, Saudi Arabia; anwar.alhazmi@gmail.com; 6 Department of Orthodontics, Faculty of Dentistry, King Abdulaziz University, Jeddah 21589, Saudi Arabia; aemasoud@kau.edu.sa

**Keywords:** bonding agents, ceramic brackets, debonding, enamel cracks, thermocycling

## Abstract

Despite the rise in popularity of ceramic braces for adults, the risk of enamel microcracks (EMCs) upon removal remains a significant drawback for both dental professionals and patients. Our study aimed to assess the effects of thermocycling, pre-curing, and co-curing techniques with different bonding agents on the enamel surface of teeth after the removal of ceramic brackets. We also examined the incidence, quantity, length, and direction of EMCs on tooth surfaces. Additionally, the adhesive remnant index (ARI) scores and orthodontic bracket bond failure modes were evaluated and compared. The study divided 40 extracted upper canine teeth into ten groups for further analysis. Two groups had intact enamel as the negative control, while the remaining groups had orthodontic ceramic brackets bonded using different bonding agents and curing techniques. Thermocycling was performed in five groups, and ARI was assessed after debonding. The study findings were statistically significant (*p* < 0.05) in demonstrating the impact of curing techniques on EMCs and debonding outcomes. Seventh-generation bonding agents resulted in complete adhesive removal (ARI = 0). The microcracks’ incidence, number, and length showed insignificant results. Differences in ARI between thermocycler and non-thermocycler samples were insignificant. Both co-curing and pre-curing techniques yielded comparable ARI results. This study highlights the importance of using advanced bonding agents to minimize enamel damage during ceramic bracket debonding.

## 1. Introduction

Ceramic brackets, also known as esthetic or clear brackets, were presented to orthodontics in the 1980s and have gained popularity due to their esthetic appeal and inconspicuousness. They offer benefits, such as biocompatibility, color stability, mechanical resistance, radiopacity, and low thermal conductivity [1,2]. However, they also have drawbacks, which include low fracture toughness, lack of ductility, high cost, and susceptibility to staining [3].

Placing ceramic brackets on the buccal surfaces, compared to placing different orthodontic brackets on various other positions, such as palatal or lingual surfaces, is an area of growing interest in orthodontic practice. This approach offers a valuable alternative for patients seeking a more discreet orthodontic solution as the appliances are more aesthetic, contributing to patient satisfaction [4].

Enamel damage and enamel microcracks (EMCs) during the debonding of ceramic brackets are major concerns for clinicians [5]. This poses a significant problem for patients seeking orthodontic treatment, as EMCs compromise enamel integrity and can lead to stains and plaque accumulation [1,6]. Enamel loss can occur during debonding, especially at the adhesive-enamel interface, resulting in enamel cracks that may spread further. Adhesive failure can happen within the resin, at various interfaces, or at different sites, influenced by factors such as tooth preparation, bonding material, and bracket debonding techniques [7].

Assessing factors related to EMCs in ceramic brackets is crucial. Different bonding agents have been developed since the introduction of orthodontic bracket bonding [8]. Light-cured materials [9] have been used for accurate bracket placement, providing a strong bond at the enamel-composite interface. Dentists often use the Adhesive Remnant Index (ARI) to measure how well the bonding material holds the bracket to the tooth. [10,11]. Ceramic brackets encounter various physical and mechanical challenges in the oral environment, such as thermal fluctuations and exposure to food and beverages. Failures can occur at the interface between adhesive materials, bonding agents, and ceramic brackets during orthodontic movement and thermal cycling, primarily due to the forces exerted by archwires. Thermal cycling, which involves exposing materials to alternating temperature variations, is used to simulate oral conditions and evaluate the durability of these materials [12,13,14].

To prevent EMCs and enamel damage, various methods for enamel surface modification and bonding have been described. Some methods aim to reduce polymerization shrinkage and enhance marginal sealing during the curing process, such as pre-curing or co-curing [15,16,17]. The choice of bonding agent type is also essential. Fifth- and seventh-generation dental agents have been widely used; older braces (etch-and-rinse or fifth-generation) use a two-step process to stick the bracket to a tooth. This involves etching the enamel (making it rough) with a separate acid solution before applying the bonding agent. Newer braces (self-etch mode or seventh-generation) are faster and simpler. They use a one-step process where the etching and bonding happen simultaneously in a single solution. This can be especially helpful for younger patients who might find the extra steps of the older method more uncomfortable [18,19].

Despite extensive research on bonding agents and curing techniques, there remains a lack of comprehensive understanding of their effects on EMC formation post-debonding of ceramic brackets. This study aimed to fill this gap by systematically evaluating the incidence, number, length, and direction of EMCs using various bonding agents and curing techniques.

## 2. Methods

### 2.1. Ethical Approval

The Research and Ethics Committee approved the study at the Faculty of Dentistry, Jazan University, Jazan City, Kingdom of Saudi Arabia; Study ID: (REC-45/07/937).

### 2.2. Study Setting and Sample

This research, conducted at King Abdulaziz University’s dental research lab in Saudi Arabia, used 40 healthy, extracted upper canine teeth. The teeth were ethically sourced from oral surgery and orthodontic clinics, following approval from Jazan University’s ethics committee and international ethical guidelines. Any teeth with cavities, developmental defects, fillings, or cracks were excluded from the study. 

The healthy maxillary canines had been extracted for various reasons, such as tooth impaction in the upper jaw, or the need to arrange crowded teeth following canine tooth extraction. Studying the anatomical characteristics of the canine tooth surface can lead to a better understanding of some of the clinical problems faced with this type of tooth and aids in finding appropriate solutions before and during orthodontic treatment.

We calculated the sample size using an online calculator (http://www.raosoft.com, accessed on 8 February 2024) (Raosoft, Inc., Seattle, WA, USA). The sample size calculation was based on the population size of 50 and 95% confidence intervals with a margin of error of 5%, which yielded a sample size of 45 participants. Thus, we selected only 40 samples, which were distributed into 10 groups with different exposures (with/without—thermocycling testing)—(n = 4/group) for this experiment. The discrepancy between the calculated sample size of 45 and the final selection of 40 specimens was due to practical considerations, including resource limitations and specimen availability. Despite this, 40 samples are considered sufficient for meaningful statistical comparisons and reliable assessment of the bonding techniques under investigation.

### 2.3. Sample Allocation, Preparation and Intervention

Canines were rinsed using tap water and then kept in a formalin (10%) solution at room temperature. The specimens were numbered and randomly assigned to one of ten groups using the Random Allocation Software (Saghaei & Kazemi, Version 1.0, Esfahan, Iran). Each group contained four specimens (n = 4), as described in the previous study [20].

### 2.4. Bonding Procedures

The selection of bonding agents was based on their common use in orthodontic practice and their differing mechanical and adhesive properties. These bonding agents were chosen for their good quality and variations in composition and bonding strength. The Dental Orthodontic Ceramic Bracket was attached to the mid portion of the buccal surface of the teeth with the help of a bracket-holding tweezer to ensure precise positioning on the buccal surface. Two different bonding agents with distinct curing methods were utilized: 1. A 5th-generation bonding agent, coltene one coat bond, SL, Switzerland, was applied with 37% phosphoric acid for etching. 2. A 7th-generation bonding agent, Kulzer Heraeus Gluma Bond Universal, along with one adhesive light-curing orthodontic cement. The brackets were gently pressed to ensure they were correctly positioned, and excess adhesive was removed before pre- and co-curing with the same device for 3 s. All samples were subsequently immersed in distilled water and kept in the ADTRL incubation machine (Incubator Universal, Oven, Memmert manufacture, Landsberger Str. 24512623 Berlin, Germany) at 37 °C for 24 h to mimic oral conditions. The next day, five of the ten groups were subjected to 5000 cycles of thermocycling (SD Mechatronic, Feldkirchen-Westerham, Germany) between 5 °C and 55 °C, with a 30 s dwell time at each temperature. All procedures were performed by the same operator.

### 2.5. Curing Techniques

The groups (3, 5, 7, and 9) and (4, 6, 8, and 10) were chosen based on the specific bonding techniques being compared in the study. Groups 3, 5, 7, and 9 involved the application of primer followed by a 3-s cure before the adhesive cement and bracket placement, while groups 4, 6, 8, and 10 utilized the direct application of primer with adhesive cement at the same time. Then, the adhesive light-curing orthodontic cement (orthocem, FGM DENTAL Group, Fort Lauderdale, FL, USA) was applied to the bracket, which was placed on the tooth. Any residual material was extracted, and then, for optimal bonding, each side of the ceramic bracket received light curing for 3 s by an H LED curing unit (CORDLESS LED, 1200 mW/cm^2^, Woodpecker, Foshan, China) to ensure proper adhesion. This “co-curing” technique cured the primer and adhesive simultaneously.

We chose orthodontic adhesive light-curing orthodontic cement (orthocem, FGM DENTAL Group, Fort Lauderdale, FL, USA) to allow the orthodontic bracket to come off without causing damage to the enamel and without leaving residue on the tooth surface.

### 2.6. Outcome Assessment

**Figure 1:**
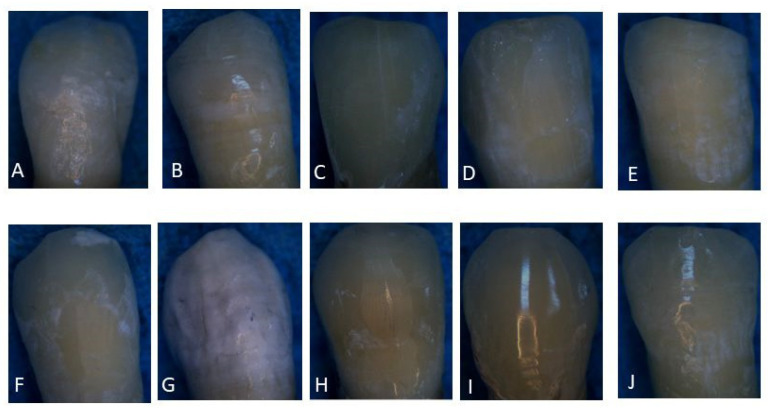
Stereomicroscope photos of enamel microcracks after the orthodontic ceramic brackets debonding in control and experimental groups (1–10) and their labeled (**A**–**J**), respectively: Group 1—Intact enamel, no brackets, no thermocycling (**A**); Group 2—Intact enamel, no brackets, with thermocycling (**B**); Group 3—Buccal enamel, ceramic bracket (5th gen, pre-curing), no thermocycling (**C**); Group 4—Buccal enamel, ceramic bracket (5th gen, co-curing), no thermocycling (**D**); Group 5—Buccal enamel, ceramic bracket (5th gen, pre-curing), with thermocycling (**E**); Group 6—Buccal enamel, ceramic bracket (5th gen, co-curing), with thermocycling (**F**); Group 7—Buccal enamel, ceramic bracket (7th gen, pre-curing), no thermocycling (**G**); Group 8—Buccal enamel, ceramic bracket (7th gen, co-curing), no thermocycling (**H**); Group 9—Buccal enamel, ceramic bracket (7th gen, pre-curing), with thermocycling (**I**); and Group 10—Buccal enamel, ceramic bracket (7th gen, co-curing), with thermocycling (**J**).

Brackets in all groups were removed using ceramic bracket debonding pliers (Carl Martin GmbH, 42657 Solingen, Germany) after both thermocycling and non-thermocycling. The excess adhesive remaining on each enamel surface was meticulously eliminated using a white polishing stone using a slow-speed handpiece to evaluate the enamel microcracks (EMCs) after orthodontic bracket debonding (Figure 1).

### 2.7. Data Management and Analysis

Researchers used software (SPSS version 22) to analyze the data. First, they checked if the numerical data followed a normal distribution (using a Kolmogorov–Smirnov test). Categorical data (without numerical values) were described using counts and percentages. To analyze these categories, a Monte Carlo exact test was used. Finally, for numerical data that was normally distributed, ANOVA (analysis of variance) tests were used to compare groups. For comparisons between just two groups with normally distributed data, they used independent t-tests. A *p*-value < 0.05 was considered statistically significant.

Enamel cracks are crucial to this study because they indicate potential damage to the tooth structure caused by different bonding techniques. Analyzing these cracks helps assess the clinical implications of each method, including the risk of post-debonding enamel damage. By understanding how different application techniques affect enamel integrity, the study aims to inform safer orthodontic practices and improve patient outcomes.

After debonding, the ceramic brackets and the enamel surface were inspected under a stereomicroscope at 70× magnification (MEIJI, EMZ-13TRD, MEIJI Techno, Saitama, Japan) to identify the failure mode. This procedure was done by a single operator only. Additionally, the adhesive on the surface was assessed using the Adhesive Remnant Index (ARI). The enamel surface condition post-debonding was classified into four grades [21].

## 3. Results

Table 1 describes the ARI between the different studied groups. In Group 3, adhesive remnants were distributed across the index categories, with 50.0% of samples showing no remnants (index 0), 25.0% with remnants classified as index 1, and 25.0% with remnants classified as index 3. Groups 7, 8, 9, and 10 demonstrated a uniform pattern, with all samples showing no adhesive remnants (index 0). There were insignificant variations in the ARI among the eight groups analyzed (*p*-value > 0.05).

Table 2 compares ARI across different groups categorized by bonding agent generation, thermocycling test, and curing technique. Regarding bonding agent generation, 43.8% of samples used fifth-generation bonding agents which showed no adhesive remnant (index 0), followed by 37.5% with index 1, 12.5% with index 2, and 6.3% with index 3. Notably, the seventh-generation bonding agents demonstrated statistically significantly better performance, with all samples (100.0%) showing no adhesive remnant (index 0). Regarding the thermocycling test, there were insignificant results observed in the ARI between samples that underwent thermocycling and those that did not. Both co-curing and pre-curing techniques demonstrated comparable results in ARI. Most samples in both groups showed no adhesive remnant (75.0% for co-curing and 68.8% for pre-curing) with insignificant results.

Table 3 shows the comparison between the different studied groups according to the presence of enamel micro-cracks. In groups 1, 2, 7, and 8, all samples showed an absence of enamel micro-cracks, with a 100.0% absence rate. Groups 3, 6, 9, and 10 had varying proportions of enamel micro-cracks, with half of the samples in each group exhibiting the presence of micro-cracks (50.0%). In groups 4 and 5, more enamel micro-cracks were present, with 75.0% of samples showing the presence of micro-cracks. There were insignificant variances in the occurrence of enamel micro-cracks (*p*-value > 0.05).

Table 4 demonstrates the contrast between different study groups according to the number and length of enamel micro-cracks. Groups 3, 4, 9, and 10 had one observed crack in 100% of samples. Group 5 had one crack in 25.0% of samples and two cracks in 75.0% of samples. Group 6 had one crack in 50.0% of samples and two cracks in the other half. There were insignificant results in the number of cracks (*p*-value > 0.05).

Regarding the length for crack #1, group 5 exhibited the most extended average length (32.45 ± 2.52), followed by group 4 (28.71 ± 5.18), and group 3 (25.94 ± 2.55). For crack #2, only group 5 and group 6 showed measurable lengths, with average lengths of 23.93 ± 3.60 and 26.69, respectively. Groups 1, 2, 7, and 8 showed a 100% absence of micro-cracks and were therefore not included in this table to highlight the results relevant to the incidence and characteristics of the cracks. The statistical analysis using ANOVA and the independent t-test revealed insignificant results in the size of cracks (*p*-value > 0.05).

**Figure 2:**
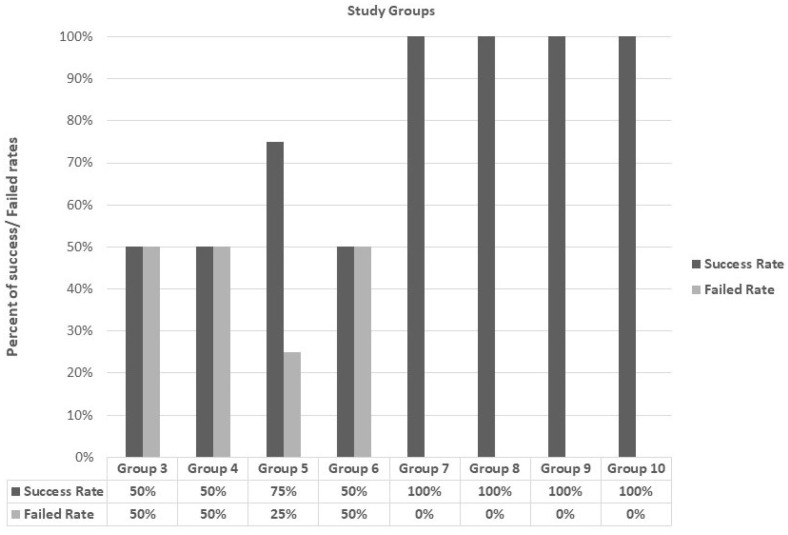
Comparison between the eight studied groups according to bond orthodontic brackets’ success after debonding: it succinctly summarizes the experimental groups (3–10) and the outcomes in terms of orthodontic bracket bond success rates after de-bonding, emphasizing the varying success rates observed across the different experimental conditions.

Figure 2 illustrates the comparison between the eight studied groups according to the success of bond orthodontic brackets after debonding. Groups 7, 8, 9, and 10 had bond orthodontic brackets success after debonding in 100% of the samples. Groups 3, 4, and 6 had bond orthodontic brackets’ success after debonding in 50.0% of samples and failed in the other half. Group 5 failed in 75.0% of samples.

**Figure 3:**
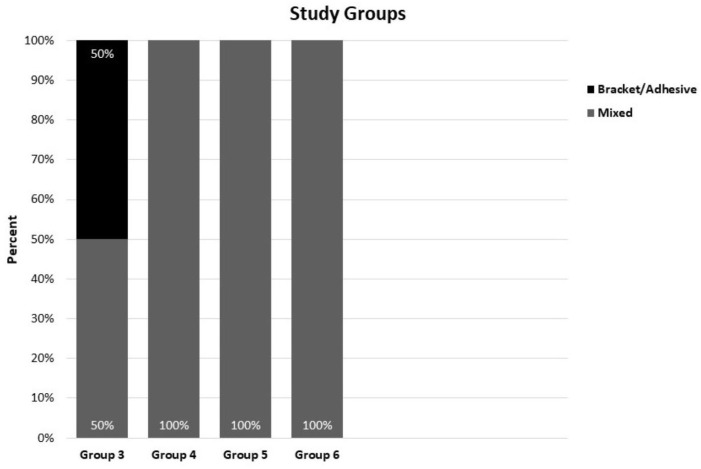
Comparison between the four studied groups according to the mode of bond orthodontic brackets failure after the debonding.

Figure 3 represents the mode of bond orthodontic brackets failure after debonding. Groups 4, 5, and 6 exhibited a mixed failure mode in 100% of samples, while group 3 exhibited a mixed failure mode in 50%. These data were obtained by analyzing the types of failures (adhesive, brackets, mixed) during the debonding process for each bonding technique applied to the groups.

## 4. Discussion

Using esthetic brackets in the treatment of adult patients has become increasingly common. With advancements in product design, ceramic brackets have shown superior performance. However, concerns regarding damage to the enamel during the debonding of ceramic brackets have led to numerous studies focusing on this issue [22]. The primary goal of any treatment approach is to minimize iatrogenic damage [23]. Therefore, reducing the occurrence of EMCs is crucial to preserve the tooth’s pre-treatment condition. Orthodontic practitioners strive to find techniques that have less adverse effects on teeth. Consequently, researchers have evaluated various factors related to the bonding procedure, including bonding agent types and different curing techniques [24].

The buccal surfaces were observed under a stereomicroscope at 70× magnification to inspect for any failure mode or bracket fractures. Successful bond orthodontic brackets after debonding are indicative of sufficient bond strength. Thus, the main challenge in rebonding brackets is restoring the bracket base to a retentive pattern without damaging the bracket itself. A failed bracket means poor bond strength and a high risk of damaging the brackets and enamel surface.

The evaluation of EMCs in different study groups revealed remarkable findings. Groups 7 and 8, which were bonded using a seventh-generation bonding agent and not subjected to thermocycling, showed a 100% absence of EMCs [25]. This suggests that these two factors may contribute to minimizing the occurrence of microcracks. In contrast, groups 3, 6, 9, and 10 exhibited varying proportions of EMCs, with half of the samples in each group showing the presence of EMCs. Groups 4 and 5 had a higher incidence of EMCs, with three-quarters of samples displaying micro-cracks. The findings emphasize the potential risk of EMCs during bracket debonding. The absence of adhesive residue in groups 7, 8, 9, and 10, despite better bond strength, warrants further investigation to confirm these preliminary observations. Most studies indicate that optimal bond strength does not necessarily preclude adhesive residue. Bishara et al. [5] observed that the occurrence of enamel damage after debonding was similar among the groups studied. Additionally, most teeth had significant adhesive remaining on the surface following debonding. In Bishara et al.’s study, forty extracted upper first premolars were stratified into two groups, each bonded with different ceramic brackets. Ten samples shattered into multiple pieces during debonding, leaving a substantial amount of resin on the tooth surface [24].

Dumbryte et al. examined the effect of different bonding protocols on enamel cracks and found that self-etching primers (similar to seventh-generation bonding agents) resulted in fewer enamel cracks compared to conventional acid etching [26]. This supports the potential benefits of newer bonding technologies. However, Zachrisson et al., in a long-term follow-up study on enamel cracks after orthodontic treatment, found that while some cracks were present immediately after debonding, these were generally not clinically significant and tended to diminish over time [27]. They did not specifically look at seventh-generation bonding agents, suggesting an area for future research.

In our study, an ARI score of 3 (indicating all adhesive was left on the tooth) was only present in group 3 (fifth-generation bonding). Groups 4 and 5 (fifth-generation bonding) also showed higher ARI scores of 1 and 2, showing partial remnants of adhesive left on the enamel surface. Interestingly, groups 7, 8, 9, and 10, subjected to seventh-generation bonding agents, had an ARI score of 0, suggesting no adhesive residue on the tooth surface, achieving a 100% success rate. This result is consistent with the literature emphasizing the efficacy of seventh-generation bonding agents in achieving the full removal of adhesives during bracket debonding. A study by Nair et al. [28] found that seventh-generation bonding agents exhibited better bond strength and easier adhesive removal compared to previous generations. Additionally, Ibrahim et al. [29] concluded that the utilization of self-etch seventh-generation bonding agents, which perform both etching and priming of the enamel simultaneously, has the potential to minimize enamel damage and reduce the residual adhesive remaining on the enamel following bracket debonding. The advancements in adhesive technology may contribute to this, simplifying the bonding protocol and eliminating errors that may occur while mixing separate components [19]. Contrarily, a study by Scribante et al. found that self-etching adhesives left more adhesive on the tooth surface compared to conventional systems [30]. This discrepancy highlights the need for further research in this area.

The lack of difference in the ARI between samples that underwent thermocycling and those that did not indicate that thermocycling did not substantially impact the adhesive amount left on the enamel surface following ceramic bracket debonding. This indicates that the bonding agents and application techniques used in the study could maintain their adhesive properties and withstand the thermal stresses imposed during thermocycling. This finding is supported by a survey by Yuasa et al. [12], which found insignificant differences in bond strength after thermocycling. Additionally, research has revealed that aging tests, such as thermocycling, do not significantly impact the mode of bracket failure when utilizing different bonding approaches. This finding suggests that enamel-adhesive and bracket-adhesive interfaces exhibit comparable strengths [29,31]. However, other studies have reported that thermal cycling can significantly reduce bond strength [32]. Hellak et al. compared various adhesive systems and found that seventh-generation bonding agents provided adequate bond strength for orthodontic purposes, although not significantly different from fifth-generation systems. This partially supports our observations [33].

Both co-curing and pre-curing techniques demonstrated comparable results in ARI, with most samples in both groups showing no adhesive remnant. This resonates with previous research comparing different application techniques, demonstrating a lack of evidence favoring the co-curing technique [34]. On the other hand, Viswanathan et al. [35] stated a considerable reduction in shear bond strength to dentin when co-cured. Therefore, it is essential to consider the specific clinical context and further investigate the optimal application technique based on individual patient needs and considerations. 

Moving forward, implementing these findings in clinical settings holds significant promise for enhancing patient care. Incorporating newer bonding agents and optimizing curing techniques according to material-specific recommendations can potentially reduce the risk of EMCs and enamel damage during orthodontic treatments. Clinicians may consider adopting pre-curing or co-curing strategies based on the material used and the patient’s enamel condition to minimize procedural risks and improve treatment outcomes.

Despite these advancements, practical limitations exist that warrant consideration. Variations in operator skill, patient compliance, and individual enamel characteristics can influence treatment outcomes. Additionally, while our study focused on ceramic surfaces, the applicability of these findings to other materials such as metal or composite brackets requires further investigation. Addressing these limitations through continued research and clinical trials will be crucial for refining treatment protocols and ensuring consistent patient care across diverse orthodontic practices.

Limitations of this study include its in vitro design, small sample size, lack of long-term evaluation, limited exploration of bracket types, absence of clinical correlation, and failure to consider potential confounding factors. In this study, we did not measure the force used for debonding. While our study provides valuable insights, these limitations may affect the generalizability of the results. Future studies with larger sample sizes and in vivo conditions are needed to validate these findings. Few studies measured the debonding forces for orthodontic brackets by different methods [35,36]. This study highlighted the significant impact of bonding agent generation and thermocycling on the occurrence of EMCs and ARI. These findings contribute to our understanding of the factors influencing enamel damage during ceramic bracket debonding and emphasize the importance of utilizing advanced bonding techniques to ensure successful and safe orthodontic treatments. By prioritizing patient safety and minimizing iatrogenic effects, orthodontic practitioners can strive to provide optimal care.

## 5. Conclusions

This in vitro study demonstrated that the choice of bonding agents and curing techniques significantly impacts the formation of enamel micro-cracks and the debonding outcomes of ceramic brackets. While the seventh-generation bonding agents provided superior performance with complete adhesive removal and minimal enamel damage, the differences in ARI scores between thermocycling and non-thermocycling groups were insignificant. Both pre-curing and co-curing techniques yielded comparable results, indicating that either method can be effectively used in clinical practice to minimize enamel damage. This study underscores the importance of using advanced bonding agents and proper curing techniques to optimize the clinical outcomes of ceramic bracket removal, thereby reducing the risk of enamel micro-cracks and enhancing overall patient satisfaction. However, further research is needed to explore the potential effects of thermocycling on bond strength and to determine the optimal application technique that may improve orthodontic bracket debonding outcomes for the buccal surface of the tooth and minimize enamel damage. 

## Figures and Tables

**Table 1:** Comparison between the different studied groups according to adhesive remnant index.

Study Groups	Adhesive Remnant Index								(^MC^ *p*-Value)
	0		1		2		3		
	No.	Total (%)	No.	%	No.	%	No.	%	
Group 3	2	4 (50.0%)	1	4 (25.0%)	0	4 (0.0%)	1	4 (25.0%)	0.228
Group 4	2	4 (50.0%)	1	4 (25.0%)	1	4 (25.0%)	0	4 (0.0%)	
Group 5	1	4 (25.0%)	2	4 (50.0%)	1	4 (25.0%)	0	4 (0.0%)	
Group 6	2	4 (50.0%)	2	4 (50.0%)	0	4 (0.0%)	0	4 (0.0%)	
Group 7	4	4 (100.0%)	0	4 (0.0%)	0	4 (0.0%)	0	4 (0.0%)	
Group 8	4	4 (100.0%)	0	4 (0.0%)	0	4 (0.0%)	0	4 (0.0%)	
Group 9	4	4 (100.0%)	0	4 (0.0%)	0	4 (0.0%)	0	4 (0.0%)	
Group 10	4	4 (100.0%)	0	4 (0.0%)	0	4 (0.0%)	0	0.0%	

MC: Monte Carlo.

**Table 2:** Comparison of adhesive remnant index of groups regarding the bonding agent generation, thermocycling test, and curing technique.

Variables		Adhesive Remnant Index								(^MC^ *p*-Value)
		0		1		2		3		
		No.	%	No.	%	No.	%	No.	%	
Bonding agent generation	5th	7	43.8%	6	37.5%	2	12.5%	1	6.3%	0.001 *
	7th	16	100.0%	0	0.0%	0	0.0%	0	0.0%	
Thermocycling test	No	12	75.0%	2	12.5%	1	6.3%	1	6.3%	0.818
	Yes	11	68.8%	4	25.0%	1	6.3%	0	0.0%	
Curing technique	Co-curing	12	75.0%	3	18.8%	1	6.3%	0	0.0%	1.000
	Pre-curing	11	68.8%	3	18.8%	1	6.3%	1	6.3%	

MC: Monte Carlo; * Significant.

**Table 3:** Comparison between the different studied groups according to the presence of enamel micro-cracks.

Study Groups	Enamel Micro-Cracks				(^MC^ *p*-Value)
	Absent		Present		
	No.	%	No.	%	
Group 1	4	100.0%	0	0.0%	0.076
Group 2	4	100.0%	0	0.0%	
Group 3	2	50.0%	2	50.0%	
Group 4	1	25.0%	3	75.0%	
Group 5	1	25.0%	3	75.0%	
Group 6	2	50.0%	2	50.0%	
Group 7	4	100.0%	0	0.0%	
Group 8	4	100.0%	0	0.0%	
Group 9	3	75.0%	1	25.0%	
Group 10	2	50.0%	2	50.0%	

MC: Monte Carlo.

**Table 4:** Comparison between the different studied groups according to the number and length of enamel micro-cracks.

		Study Groups						*p*-Value
		Group 3	Group 4	Group 5	Group 6	Group 9	Group 10	
Number of cracks	1	2 (100.0%)	3 (100.0%)	1 (25.0%)	1 (50.0%)	1 (100.0%)	2 (100.0%)	0.441 ^MC^
	2	0 (0.0%)	0 (0.0%)	2 (75.0%)	1 (50.0%)	0 (0.0%)	0 (0.0%)	
Length of cracks (mm)	Crack 1	25.94 ± 2.55	28.71 ± 5.18	32.45 ± 2.52	22.46 ± 5.36	24.12	24.02 ± 5.60	0.239 ^AN^
	Crack 2	-	-	23.93 ± 3.60	26.69	-	-	0.645 ^T^

Values are presented as number (%) or mean ± SD. MC: Monte Carlo; AN: ANOVA and T: Independent *t*-test. N.B.: The direction of cracks was vertical in 100% of teeth, which developed micro cracks.

## Data Availability

The raw data supporting the conclusions of this article will be made available by the authors upon request.
